# Transcriptome analysis of the molecular mechanism underlying immunity- and reproduction trade-off in *Locusta migratoria* infected by *Micrococcus luteus*

**DOI:** 10.1371/journal.pone.0211605

**Published:** 2019-08-14

**Authors:** Shaohua Wang, Xiaojun Liu, Zhiyong Xia, Guoqiang Xie, Bin Tang, Shigui Wang

**Affiliations:** Hangzhou Key Laboratory of Animal Adaptation and Evolution, College of Life and Environmental Sciences, Hangzhou Normal University, Hangzhou, Zhejiang, China; Chinese Academy of Agricultural Sciences Institute of Plant Protection, CHINA

## Abstract

Immune response and reproductive success are two vital energy-consuming processes in living organisms. However, it is still unclear which process is prioritized when both are required. Therefore, the present study was designed to examine this question arising for one of the world’s most destructive agricultural pests, the migratory locust, *Locusta migratoria*. Transcripts from the ovaries and fat bodies of newly emerged locusts were analyzed, using RNA-seq based transcriptome and qualitative real-time PCR, at 4 h and 6 d after being infected with the gram-positive bacteria *Micrococcus luteus*. Changes in the main biological pathways involved in reproduction and immunization were analyzed using bioinformatics. After 4 h of infection, 348 and 133 transcripts were up- and down-regulated, respectively, whereas 5699 and 44 transcripts were up- and down-regulated, respectively, at 6 d after infection. Moreover, KEGG analysis indicated that vital pathways related with immunity and reproduction, such as Insulin resistance, FoxO signaling, Lysosome, mTOR signaling, and Toll-like receptor signaling pathways were up-regulated. Among the differentially expressed genes, 22 and 17 were related to immunity and reproduction, respectively. The expression levels of *PPO1* and *antimicrobial peptide defensin 3* were increased (log_2_FC = 5.93 and 6.75, respectively), whereas those of *VgA* and *VgB* were reduced (log_2_FC = -17.82 and -18.13, respectively). These results indicated that locust allocate energy and resources to maintain their own survival by increasing immune response when dealing with both immune and reproductive processes. The present study provides the first report of expression levels for genes related with reproduction and immunity in locusts, thereby providing a reference for future studies, as well as theoretical guidance for investigations of locust control.

## Introduction

Insects are one of the most successful group of animals on earth, owing, at least in part, to the effectiveness of their immune response to microbial invasion, which include both humoral and cellular immunity. Humoral immunity mainly deals with the synthesis of antimicrobial peptides (AMPs) in the fat body and is primarily based on the Toll and Imd signaling pathways, which play important roles in AMP expression, as well as on the interaction between the two pathways [1–2]. Meanwhile, cellular immunity involves hemocyte-mediated phagocytosis, encapsulation, and nodulation. The melanization of macroparasites triggered by hemocytes participating in phagocytosis or phenoloxidase cascade activation are hallmarks of cellular response [3]. Furthermore, the JNK and JAK/STAT pathways also contribute to the immune response [4]. During these immune processes, insects must invest energy and resources to survive against a variety of pathogens.

Recently, the reproduction process of female insects has been thoroughly studied as an important target for pest control [5]. The process is mainly regulated by juvenile hormones (JH), ecdysteroids, and nutritional signaling pathways, which differs on insect species owing to differences in reproductive strategies [6]. In most insect species, the central part of female reproduction system is vitellogenesis, which involves the production of both vitellogenin (Vg) and other yolk protein precursors (YPPs), followed by the internalization of YPPs by maturing oocytes *via* receptor-mediated endocytosis [7]. Depending on tissue type, sex, and developmental stage, Vg is synthesized extra-ovarially by the fat body, secreted into the hemolymph, and then sequestered by competent oocytes *via* receptor-mediated endocytosis [7]. Vitellogenin is stored in a crystalline form, as vitelline, after being incorporated into oocytes, where it functions as a reserve food source for future embryos [8]. The reproductive process may affected by energy metabolism, nutrient metabolism, and fitness value. In *Locusta migratoria* (Orthoptera), female reproduction is governed by JHs, which contribute to vitellogenesis and oocyte maturation [9].

Although both reproduction and the ability to survive pathogenesis are essential functions, the evolution of life history is a matter of optimization, rather than maximization, so that organisms must divide their limited energy and nutritional resources among a variety of life processes. The trade-off between female insect reproduction and immunity has been reported in recent years [10]. Because both immunity and reproduction are physiological energy-consuming processes- and increases reproduction effort reduces immune ability, whereas immune system activation reduces reproductive output [11]. Identifying the genes that are involved in both reproduction and immunity during trade-off will enhance understanding of the internal regulatory mechanism underlying these processes, and also to identify the best target for biological control of insect pests.

The locust is one of the most important agricultural pests worldwide. However, little research has been conducted on the trade-off between immunity and reproduction in locusts and the genes involved. In the present study, the gram-positive bacteria *Micrococcus luteus (M*. *luteus)* was used to infect newly emerged locusts. The fat body and ovary, which are the main organs involved in immunity and reproduction, were dissected at 4 h and 6 d after parasitism. Differentially expressed genes (DEGs) analysis based on next-generation RNA-seq technology provides extensive data, with enormous depth and coverage, thereby allowing the global analysis of a parasitized host’s transcription profile. To identify immunity- and reproduction-related genes that were differentially up- or down-regulated in the infected locusts, the present study compared the fat body and ovary transcripts of (1) the control and infected locusts at 4 h after treatment, (2) the control and infected locusts at 6 d after treatment, (3) the control locusts at 4 h and 6 d after treatment, and (4) the infected locusts at 4 h and 6 d after treatment. Bioinformatic analysis and qRT-PCR verification were used to predict and explore genes involved in immunity and reproduction, as well as to identify the molecular mechanisms underlying energy and resources trade-off in *L*. *migratoria*.

## Materials and methods

### Insect rearing and experimental treatments

Eggs were collected from *L*. *migratoria* that were maintained in the insect laboratory at Hang Zhou Normal University (Zhejiang Province, China). The locust models were established by rearing the insects in each well-ventilated cage (50 × 50 × 50 cm) at densities of 200–300 insects per cage. The insects were reared on a 16 h photoperiod at 30 ± 2°C and fed on fresh greenhouse-grown wheat seedlings [12–13]. Female locusts were collected and subject to parasite treatments within 12 hours after eclosion. The treated group was infected with the bacterial pathogen *M*. *luteus* (American Type Culture Collection, Manassas, VA, USA) which had been cultured to the logarithmic phase, according to the manufacturer’s instructions by injecting the pronotum of each locust with 10 μl of bacterial culture (0.9 optical density at 600 nm) using a nanofil syringe (Shenggong, Shanghai, China). Meanwhile, the pronotum of each locust in the control group was stabbed using an alcohol-sterilized needle. For both the treatment and control groups, the wounding site of each insect was sealed using petroleum jelly to prevent exogenous natural infection. A diet of wheat seedlings and wheat bran was supplied in sufficient, but not excessive, amounts. Fat bodies and ovaries were collected from females in each group at 4 h and 6 d after injection, respectively, with two biological replicates per group. Furthermore, 30 ovaries from females in each group were dissected and weighed.

### cDNA library construction and high-throughput transcriptome sequencing

Trizol (Invitrogen, Los Angeles, CA, USA) was used to extract total mRNA from mixed tissue samples (fat body and ovary) taken from individual insects of the control and treated groups at each time point, following the manufacturer’s procedure. The quantity and purity of the total RNA were measured using a Bioanalyzer 2100 and RNA 6000 Nano LabChip Kit (RIN>7.0; Agilent, CA, USA). Poly(A) mRNA was isolated from ~10 μg of each total RNA sample using poly-T oligos attached to magnetic beads (Thermo-fisher, MA, USA). The isolated mRNA was fragmented using divalent cations under elevated temperature and then used to construct a cDNA library, following the protocol for the Illumina RNA ligation-based method (Illumina, San Diego, CA, USA). Briefly, the fragmented RNA was dephosphorylated at the 3' end using phosphatase and phosphorylated at the 5' end using polynucleotide kinase. These samples were purified using the RNeasy MinElute Kit (Qiagen, Dusseldorf, Germany), following the manufacturer’s instructions, ligated to a pre-adenylated 3' adapter, which enabled the subsequent ligation of a 5' adapter, and then subjected to both reverse transcription and PCR. The average insert size for the paired-end libraries was 300 bp (±50 bp). Finally, the library was subjected to single-end sequencing using an Illumina Hiseq 2000 by LC Sciences (Houston, TX, USA), following the vendor's recommended protocol.

### Bioinformatic analysis

Raw data was filtered using Trimommatic software with default parameters. The clean reads that were mapped to the *L*. *migratoria* genome database (http://locustmine.org/index.html) were annotated using Locust Base-locust genome data, GO, KEGG, Nr, Nt, and Swissport. The number of perfect clean reads corresponding to each gene was calculated and normalized to the number of Reads Per Kilobase of exon model per Million mapped reads (RPKM) [14]. Based on expression levels, DEGs were identified, using a P-value of ≤0.05 and log_2_ fold-change (log_2_FC) of ≥1. Gene ontology (GO) analysis (ftp://ftp.ncbi.nih.gov/gene/DATA/gene2go.gz) was conducted for functional classification of the DEGs, and pathway analysis was performed using KEGG (http://www.genome.jp/kegg).

### Quantitative real-time PCR analysis

To verify the RNA-seq data fifteen genes were selected. Primer 5.0 was used to identify appropriate primers (S1 Table), and the expression levels of the selected genes were normalized using the expression of actin. Quantitative real-time PCR (qRT-PCR) was performed using a Bio-Rad CFX96 Real-Time PCR Detection system (Bio-Rad, CA, USA) and Premix Ex Taq (SYBR Green) reagents (Takara, Dalian, China). The 20-μl qRT-PCR reactions were subject to a thermal profile of 95°C for 3 min and then 40 cycles of 95°C for 10 s and 60°C for 30 s. The thermal melting profile was assessed using a final PCR cycle of 95°C for 30 s, with temperature increasing constantly from 60 to 95°C. Relative gene expression levels were calculated using the 2^–ΔΔCT^ method, with three replicates per sample.

## Results

### Changes of ovaries in infected group and RNA-seq library analysis

**Fig 1:**
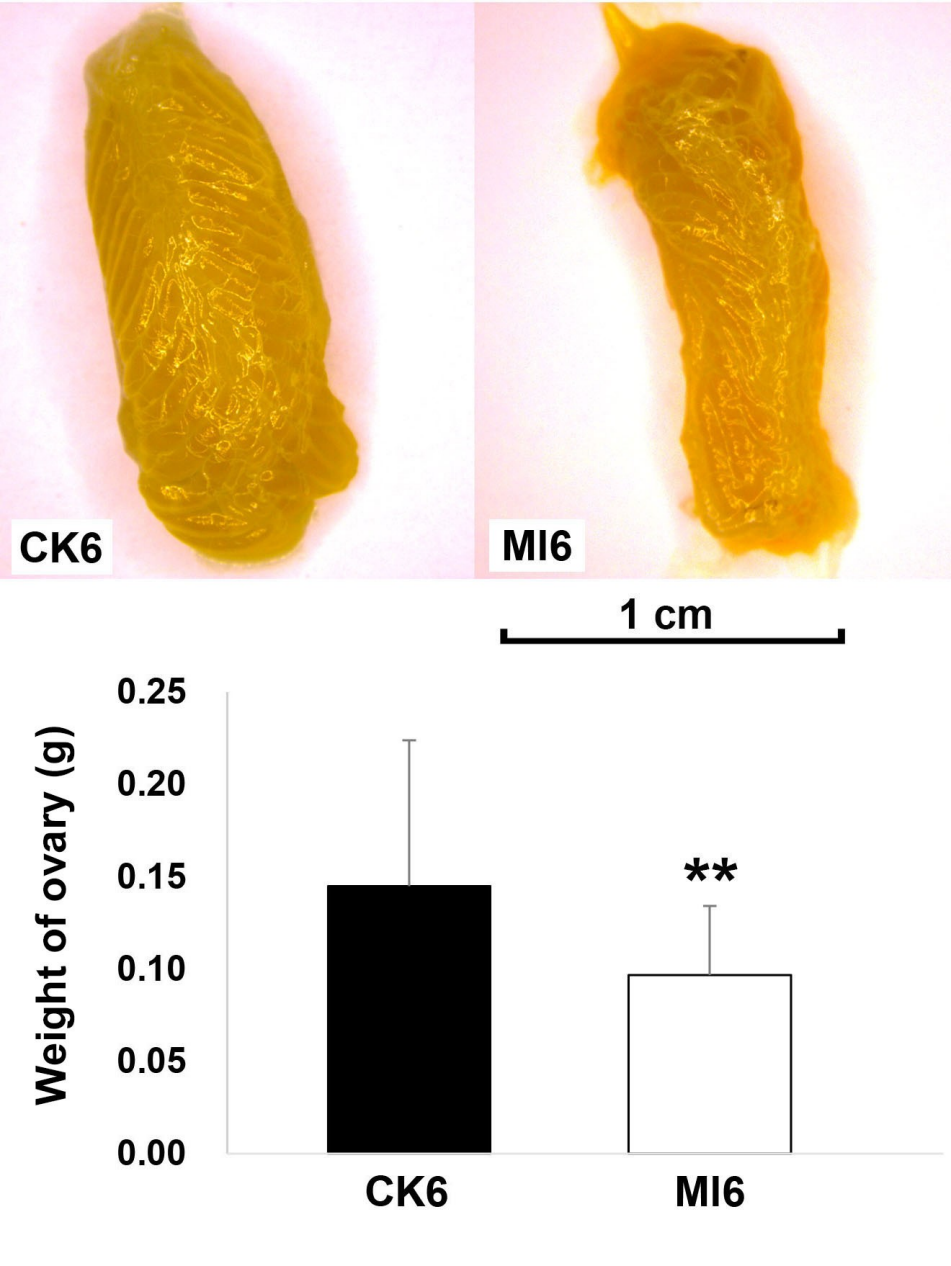
Comparison of ovary weight between control and *M*. *luteus* infected group. Ml6 indicated the *M*. *luteus* treated group at 6 d after infection and CK6 indicated its control group which infected by *M*. *luteus* culture medium only.

We compared the weight of ovaries of infected group with control group. The weight of ovaries in infected group was significantly lighter than in control group (Fig 1). In RNA-seq experiment, single end reads has been obtained with the quality high enough for the gene expression analysis subsequently (Table 1). In order to investigate the patterns of gene expression related to locust immunity and reproduction, we analyzed control and infected groups at two time points, with two biological replicates each (eight samples in total). The *L*. *migratoria*, Nr, Nt, SwissProt, GO, and KEGG databases were used to annotate the transcripts (Table 2). Most of the mRNAs had low RPKM values, typically between 30 and 80 (Table 3).

**Table 1:** Overview of sequencing data.

Sample	Raw reads	Clean reads	Clean bases	Error(%)	Q20(%)	Q30(%)	GC(%)
CK4A	14583856	14573380	0.73G	0.02	99.37	97.67	43.19
CK4B	15370335	15359428	0.77G	0.02	99.41	97.87	42.50
CK6A	18789172	18782102	0.94G	0.03	99.37	97.69	49.17
CK6B	21062414	21058652	1.05G	0.02	99.35	97.57	50.20
Ml4A	12436735	12228854	1.22G	0.03	97.90	93.43	43.17
Ml4B	10058876	9885731	0.99G	0.02	98.29	94.36	43.88
Ml6A	14433467	14199926	1.42G	0.03	97.88	93.30	43.62
Ml6B	9681097	9492635	0.95G	0.02	98.30	94.42	43.68

Note: Sample: sample name; Raw Reads: four lines as a unit to statistic the sequence numbers of each document; Clean reads: filtered raw reads; Clean bases: number of sequences×the length of sequence then turn to G; Error: mistake ration of base pairs; Q20、Q30:The percentage of bases whose Phred value is greater than 20,30 in all base pairs; GC content: The percentage of G and C in all base pairs.

**Table 2:** Annotation of identified genes.

Database	Gene number	Percentage (%)
GO	4778	29
KEGG	2984	18.1
NR	12555	76.2
NT	14672	89.1
Swiss-prot	9436	57.3
All genes	16472	100

**Table 3:** RPKM expression analysis.

Sample	Exp gene	Min.	Median	Mean	Max.	Sd.	Sum.
CK4A	12888	0.01	11.98	80.75	104051.71	1035.33	1040762
CK4B	13862	0.01	21.79	64.36	15116.55	208.81	892134.6
CK6A	12670	0.01	3.25	31.37	66286.24	848.92	397499.3
CK6B	11877	0.01	1.56	30.69	72345.81	945.36	364532.7
Ml4A	14121	0.01	20.69	64.31	3600.63	144.12	908109.6
Ml4B	14003	0.01	20.77	64.68	2376.94	144.4	905717.2
Ml6A	14411	0.01	19.05	66.32	17919.79	251.71	955735.4
Ml6B	14247	0.01	20.04	67.31	7496.22	195.61	958994.9

Note: Sample: sample name; Exp gene: genes identified in each sample; Min: minimum expression value of all genes in each sample; Median: median expression value of all genes in each sample; Mean: average expression value of all genes in each sample; Max: maximum expression value of all genes in each sample; Sd: variance of expression value of all genes in each sample; Sum: total expression value of all genes in each sample.

### DEGs analysis

**Fig 2:**
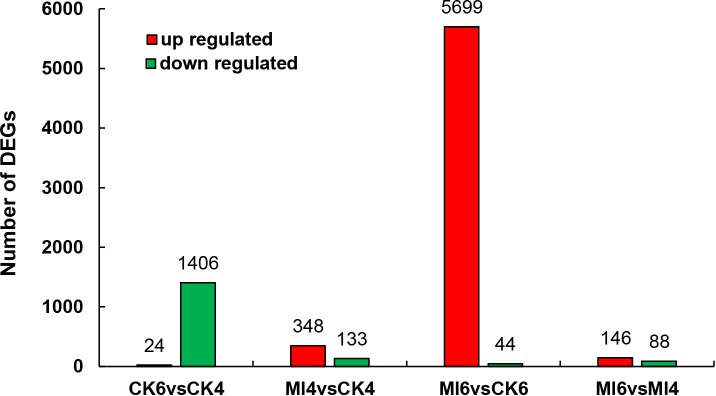
The DEGs induced by *M*. *luteus* infection. The Y axis indicates the number of DEGs. “CK4” indicates the control group at 4 h after *M*. *luteus* culture medium injection, “CK6” indicates the control group at 6 d after *M*. *luteus* culture medium injection, “Ml4” indicates the treated group at 4 h after *M*. *luteus* infection, “Ml6” indicates the treated group at 6 d after *M*. *luteus* infection. Red and green bar indicate up- and down-regulated genes, respectively.

**Fig 3:**
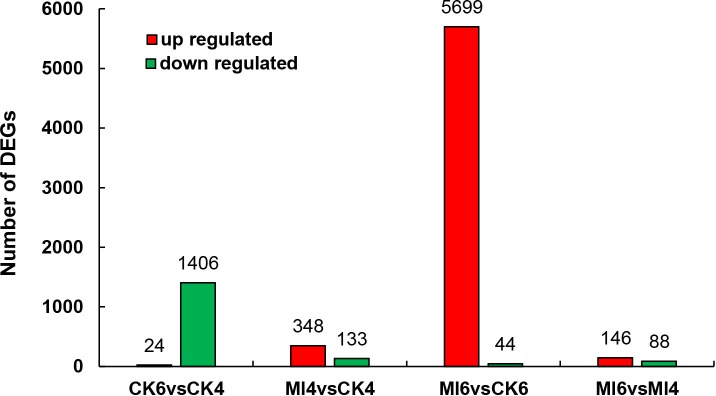
Clustering of differentially expressed genes.

Firstly, the infected and control libraries for each time point were compared (Ml4 vs CK4 and Ml6 vs CK6) to identify genes that are differentially expressed at different times of infection. At 4 h after treatment, 348 and 133 genes were up- and down-regulated, respectively, in the infected group (S2 Table) and at 6 d after treatment, 5699 and 44 were up- and down-regulated (S3 Table). Secondly, the times points for the control and infected libraries were compared (CK6 vs CK4 and Ml6 vs Ml4) to identify genes that are differentially expressed during infection. For the control group, 24 and 1406 genes were up- and down-regulated, respectively, at 6 d after treatment, when compared to 4 h after treatment (S4 Table), and for the treatment group, 146 and 48 were up- and down-regulated (S5 Table). The number of the DEGs was summarized as columnar (Fig 2). All the DEGs were clustered to produce heat maps, the two replications of controls and treatments were clearly divided into two main clusters (Fig 3).

### GO and KEGG analysis

**Fig 4:**
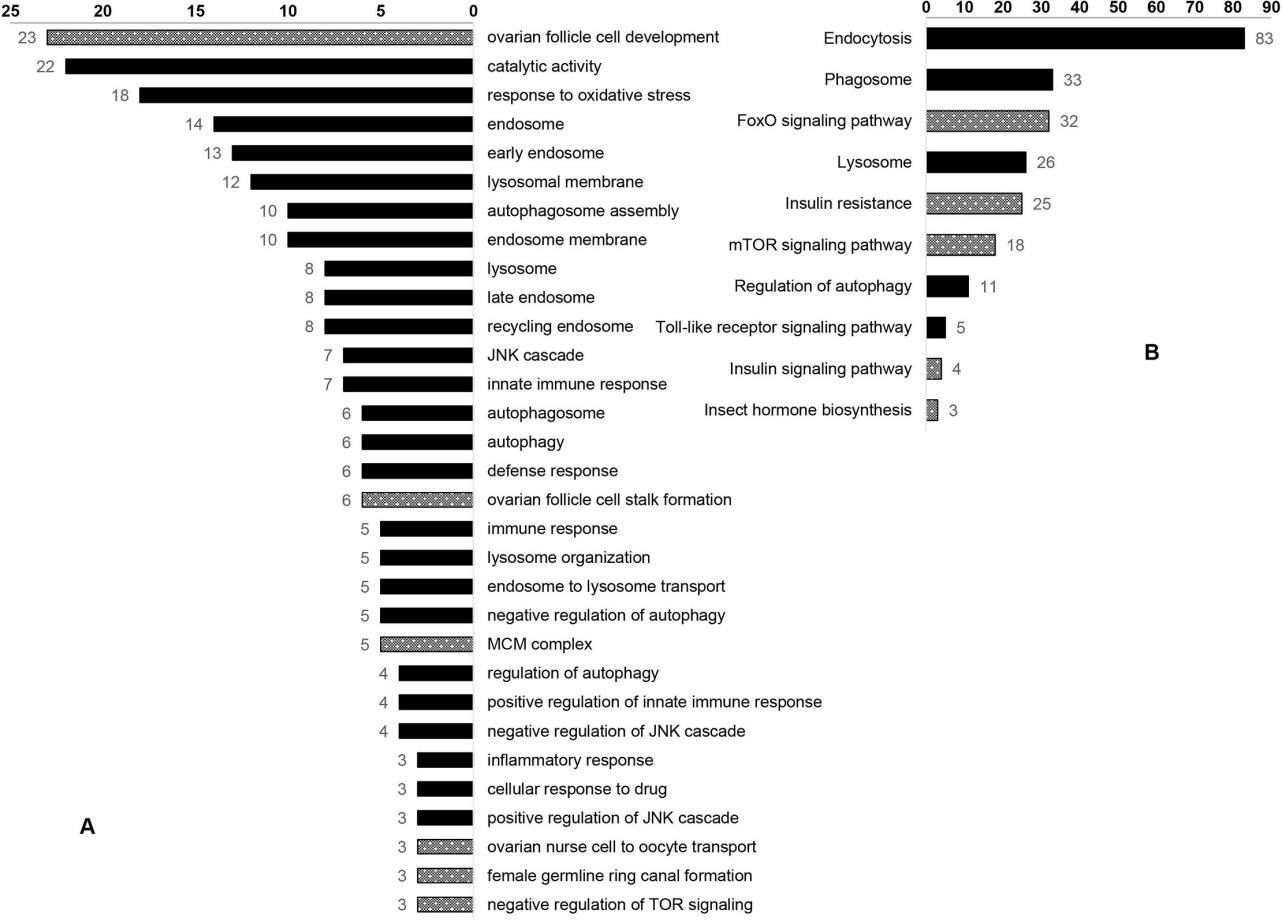
GO and KEGG terms for immunity- and reproduction-related genes that were differentially expressed among treatment groups at 6 d after infection.

**Fig 5:**
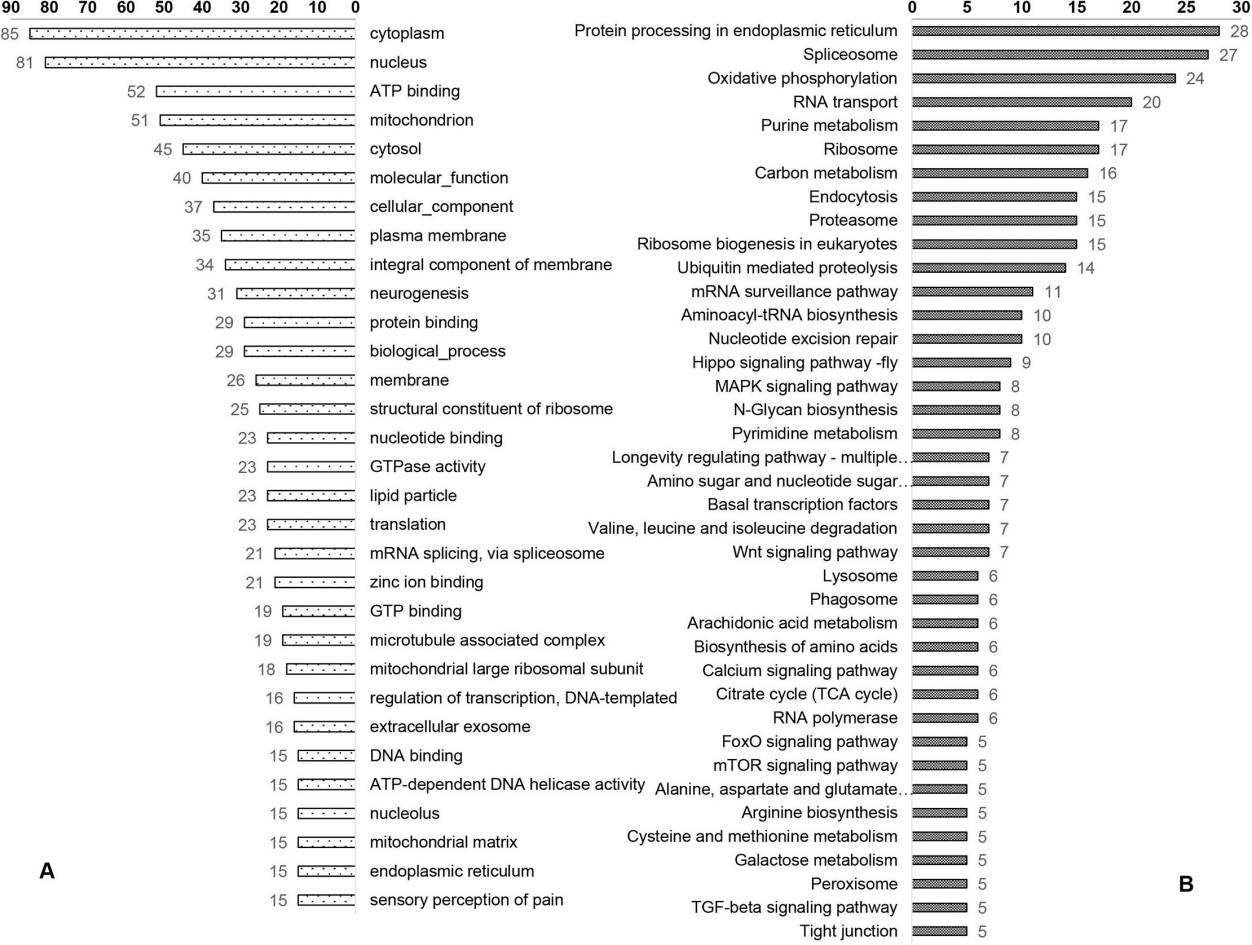
GO and KEGG terms for immunity- and reproduction-related genes that were differentially expressed in the control group at 4 h and 6 d after infection.

For the up-regulated GO terms related to immunity (Fig 4A, S6 Table), the most frequently mapped transcripts were ‘catalytic activity’ and ‘response to oxidative stresses.’ In addition, a large number of transcripts were also mapped to functional groups related to fighting against external infections, such as ‘Lysosome’ and ‘Endosome’. ‘Immunity signal JNK cascade,’ ‘innate immune response,’ ‘defense response,’ ‘immune response,’ and ‘inflammatory response’ were represented by seven, seven, six, five, and three transcripts, respectively. For up-regulated GO terms related to reproduction, six GO terms were significantly changed, including ‘ovarian follicle cell development,’ ‘ovarian follicle cell stalk formation,’ ‘MCM complex,’ ‘ovarian nurse cell to oocyte transport,’ ‘female germline ring canal formation,’ and ‘negative regulation of TOR signaling.’ For GO analysis of the transcripts up-regulated at 6 d in the control group, most of the DEGs were related to genes involved in individual growth (Fig 5A). However, when comparing transcripts from the control and infected groups at 4 h after treatment and when comparing transcripts from the infected groups at 4 h and 6 d after treatment, none of the significantly changed GO terms were directly related to immunity or reproduction.

**Fig 6:**
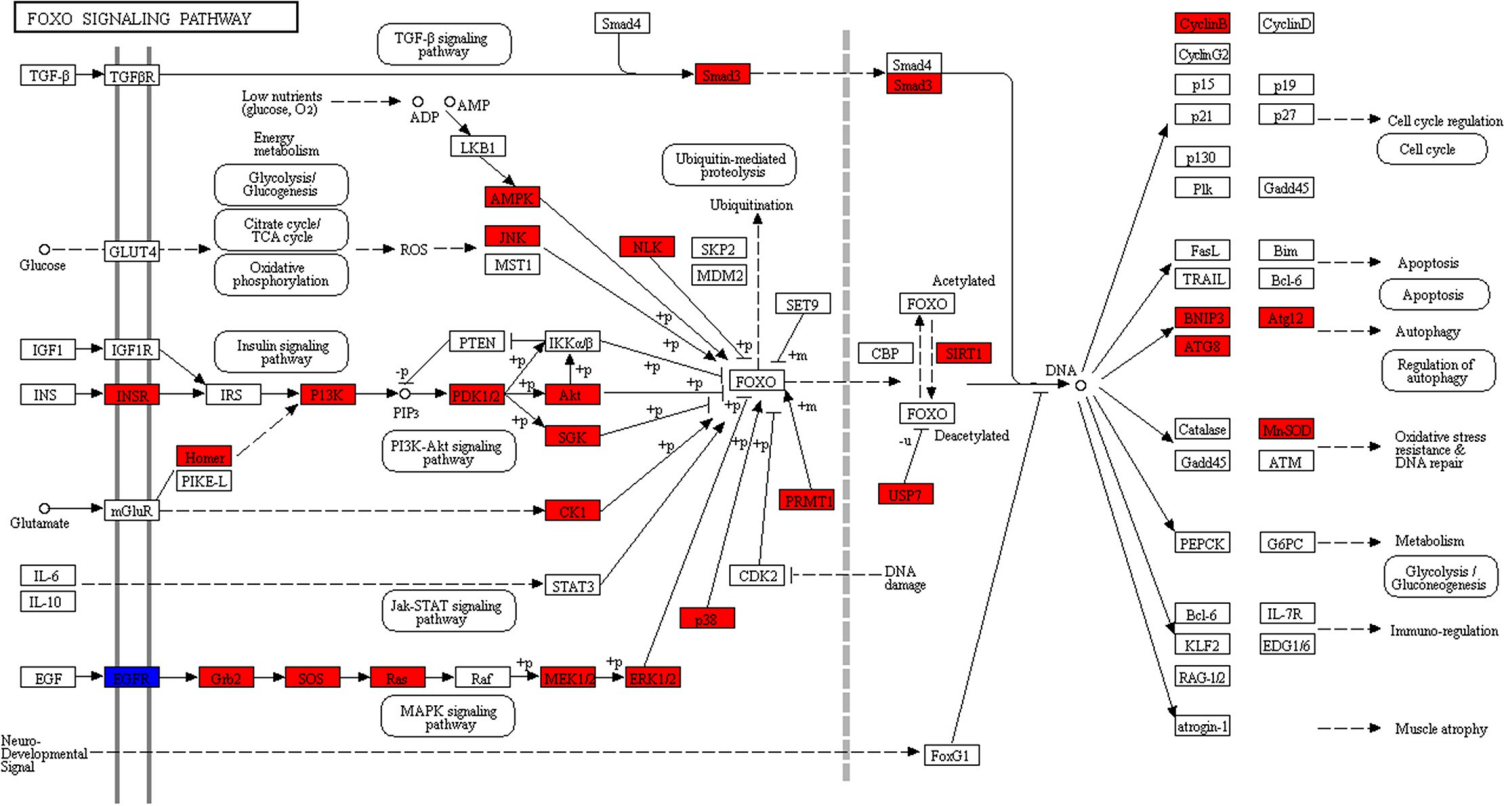
FoxO signaling pathway. Red indicates significantly up-regulated transcripts.

**Fig 7:**
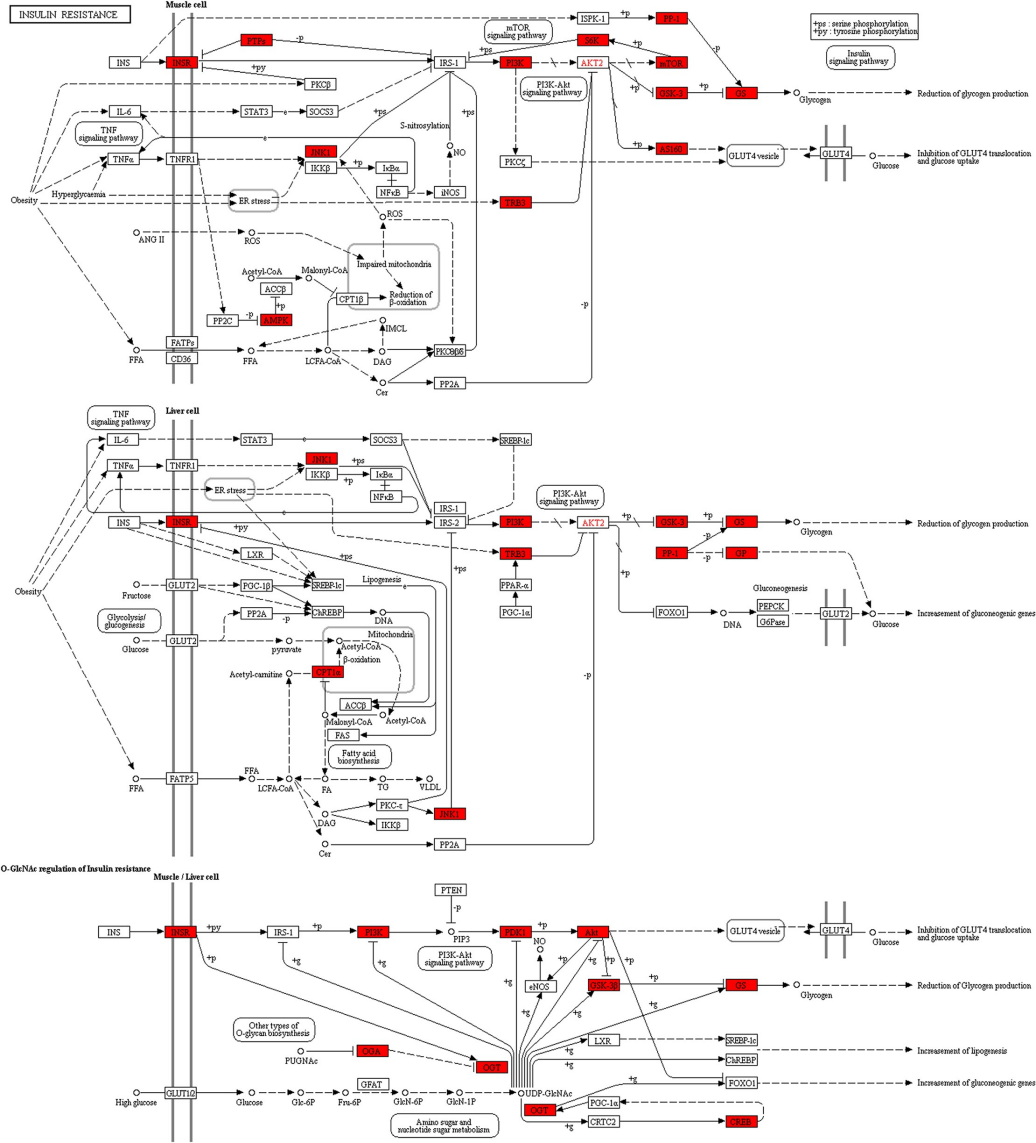
Insulin resistance pathway. Red indicates significantly up-regulated transcripts.

For the up-regulated transcripts mapped pathway (Fig 4B, S7 Table), ten of them were involved in immunity and reproduction. The screening criteria used to determine the number of mapped transcripts was more than three: ‘Endocytosis,’ ‘Phagosome,’ ‘FoxO signaling pathway’ (Fig 6), ‘Lysosome’ (S1 Fig), ‘Insulin resistance’ (Fig 7), ‘mTOR signaling pathway’ (S2 Fig), ‘Regulation of autophagy,’ ‘Toll-like receptor signaling pathway,’ ‘Insulin signaling pathway,’ and ‘Insect hormone biosynthesis.’ At 4 h after treatment, the ‘Phagosome’ pathway (S8 Table) was up-regulated in the infected group. In the control group, five down-regulated pathways were identified as involved in immunity and reproduction, including ‘Endocytosis,’ ‘Lysosome,’ ‘Phagosome,’ ‘FoxO signaling pathway,’ ‘mTOR signaling pathway.’ (Fig 5B, S9 Table). In the infected group, no pathways related to immune or reproductions were differentially expressed at 6 d after treatments, when compared to 4 h after treatment (S10 Table).

### Immunity- and reproduction-related transcripts

The GO and KEGG analyses failed to identify any significant differences between the control and infected groups at 4 h after treatment. Therefore, in searching for genes involved in reproduction and immunity, mainly we have selected significantly expressed genes in Ml6 vs CK6 group.

Of the transcripts up-regulated at 6 d after treatment, 22 were annotated as immunity-related genes (Table 4), and *defensin 3* and *PPO1* were increased the most (log_2_FC = 5.93 and Inf, respectively; where, ‘Inf’ indicates that the expression of the transcript in the control group was extremely low, so that the resulting ratio approached infinity). Two genes participated in Toll signaling, namely, *Toll-9 receptor* and *Toll-like receptor 6*, were up-regulated in the infected group, as were *AP-1 complex subunit gamma-1*, *AP-2 complex subunit alpha*, and *AP-3 complex subunit mu-1*, *serine protease inhibitor 27A*, and *serine protease inhibitor 4-like protein*.

**Table 4:** Differentially changed genes related to immunity and reproduction.

Accession	Log_2_FC	P-value	Annotation
**Immunity Related**			
LOCMI01321-m1	Inf	0.03	defensin 3
LOCMI17352-m1	5.93	0.03	pro-phenoloxidase 1
LOCMI07022-m1	5.88	0.01	lipase 3
LOCMI08957-m1	5.65	0.01	Caspase
LOCMI09550-m1	4.91	0.05	multidrug resistance-associated protein 1
LOCMI06608-m1	4.61	0.04	Toll-9 receptor
LOCMI11959-m1	4.49	0.04	serine protease inhibitor 27A
LOCMI04359-m1	4.22	0.05	serine protease inhibitor 4-like protein
LOCMI07530-m1	4.1	0	I-type lysozyme
LOCMI15220-m1	3.84	0	septin 6
LOCMI05743-m1	3.65	0.01	AP-2 complex subunit alpha
LOCMI16411-m1	3.51	0.03	IMD-like protein
LOCMI12803-m1	3.2	0	stress-activated protein kinase JNK
LOCMI06913-m1	2.94	0.01	AP-1 complex subunit gamma-1
LOCMI10597-m1	2.39	0.01	Cyclic AMP-dependent transcription factor ATF-6 beta
LOCMI16441-m1	2.12	0.04	Toll-like receptor 6
LOCMI16401-m1	1.98	0.03	AP-3 complex subunit mu-1
LOCMI09105-m1	1.96	0.04	lysosomal alpha-glucosidase-like
LOCMI03672-m1	1.9	0.05	allergen
LOCMI08404-m1	1.67	0.02	lectin 1
LOCMI14730-m1	1.4	0.03	toll interacting protein
LOCMI16384-m1	-5.13	0.03	proteinase inhibitor serpin	
**Reproduction-Related**			
LOCMI17613-m1	7.25	0.01	juvenile hormone acid methyltransferase
LOCMI16603-m1	6.75	0.05	Vitellogenin receptor
LOCMI07370-m1	6.45	0.01	insulin-like receptor
LOCMI11173-m1	5.6	0	MCM7
LOCMI16330-m1	5.42	0	MCM4
LOCMI17110-m1	5.05	0.01	protein kinase C
LOCMI16691-m1	4.27	0.04	juvenile hormone epoxide hydrolase-like protein 5
LOCMI17227-m1	4.14	0.01	argonaute 1
LOCMI15502-m1	4.06	0.01	fem-1 homolog A-like protein
LOCMI17278-m1	4.05	0.01	serine/threonine-protein kinase NLK
LOCMI10714-m1	3.99	0.01	juvenile hormone-regulated gene
LOCMI13916-m1	3.7	0.04	Hormone-sensitive lipase
LOCMI12953-m1	3.4	0.01	calcium/calmodulin-dependent protein kinase II
LOCMI09508-m1	3.3	0.01	Protein Mo25
LOCMI16380-m1	2.73	0.02	target of rapamycin
LOCMI12128-m1	-17.82	0.03	vitellogenin A
LOCMI12129-m1	-18.13	0.02	vitellogenin B

Seventeen reproduction-related transcripts were up-regulated, whereas both *VgA* and *VgB* were sharply down-regulated (log2FC = -17.82 and -18.13, respectively). Several genes related to JH metabolism, such as *juvenile hormone acid methyltransferase*, *juvenile hormone epoxide hydrolase-like protein 5*, and *juvenile hormone-regulated gene*, were also up-regulated in the infected group, as were *insulin metabolism related gene* and *insulin-like receptor*.

### qRT-PCR validation of DEGs

**Fig 8:**
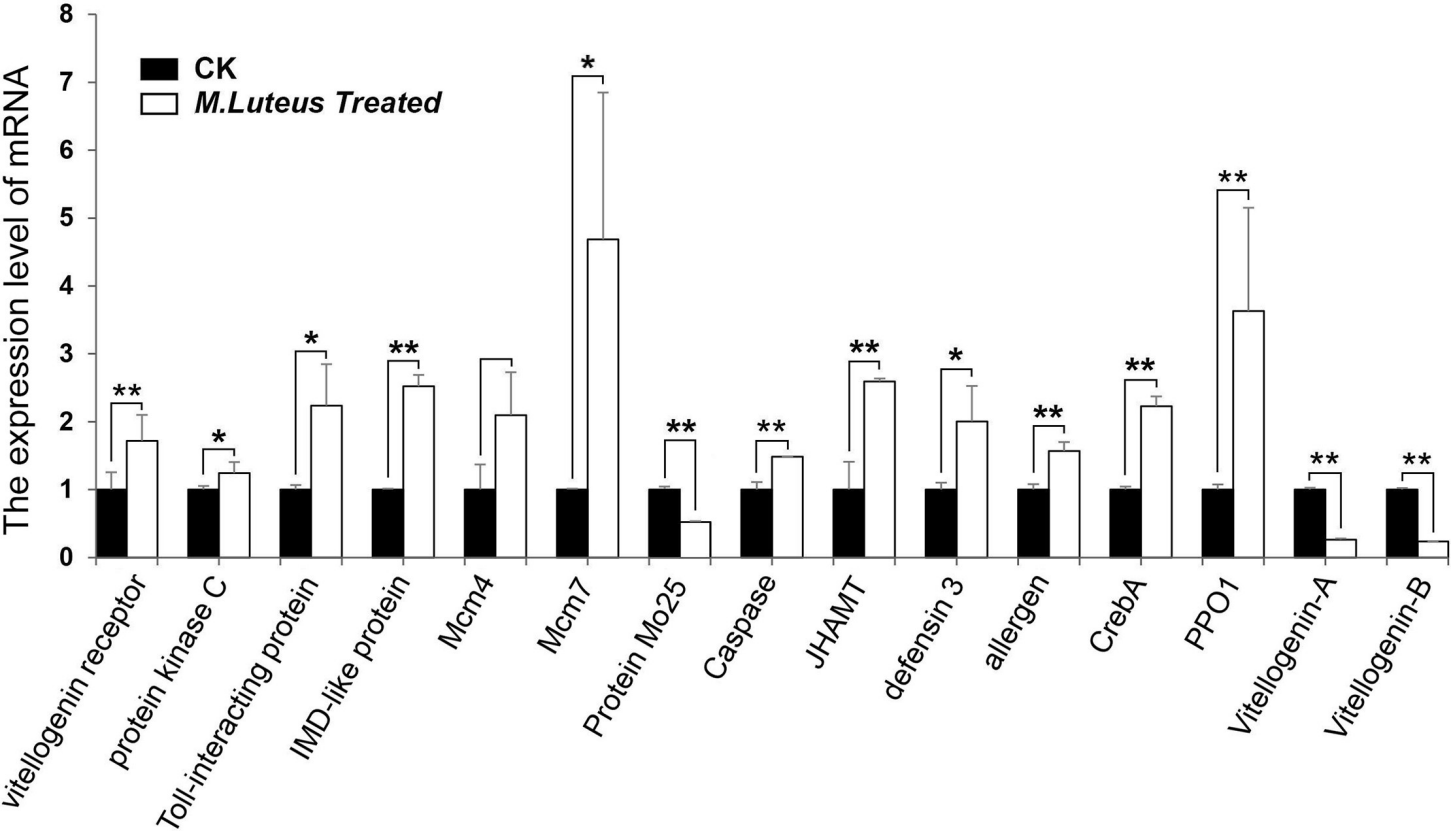
Quantitative RT-PCR analysis of 15 DEGs at 6 d after infection. The expression level of the DEGs in control group was normalized as “1”. “CK6” indicates the control group at 6 d after *M*. *luteus* culture medium injection, “Ml6” indicates the treated group at 6 d after *M*. *luteus* infection. “*” means P<0.05, “**” means P<0.01.

Fifteen DEGs involved in either immunity or reproduction were randomly selected to verify the accuracy of the RNA-seq data using qRT-PCR. The qRT-PCR results validated the expression of thirteen genes (Fig 8). The differentially expression of one was not significant, while another was down-regulated. The overall trends of most of the genes were consistent, which indicated that the DGE results were reliable enough to interpret scientific problems we focused in this present study (Table 5).

**Table 5:** Gene models verified using qRT-PCR.

Genes	DGE Log_2_FC	qRT-PCR Log_2_FC	Concordant
LOCMI16603-m1	6.75	0.78	yes
LOCMI17110-m1	5.05	0.32	yes
LOCMI14730-m1	1.4	1.16	yes
LOCMI16411-m1	2.12	1.33	yes
LOCMI16330-m1	5.42	1.07	no
LOCMI11173-m1	5.6	2.23	yes
LOCMI09508-m1	3.3	1.07	no
LOCMI08957-m1	5.65	-1.07	yes
LOCMI17613-m1	7.25	1.38	yes
LOCMI01321-m1	Inf	1	yes
LOCMI03672-m1	1.9	0.65	yes
LOCMI10597-m1	2.39	1.16	Yes
LOCMI17352-m1	5.93	1.86	Yes
LOCMI12128-m1	-17.82	-1.93	Yes
LOCMI12129-m1	-18.13	-2.09	Yes

## Discussion

Resource trade-offs between immunity and reproduction may arise as a consequence of direct physiological conflicts between the two processes [10]. In the present study, the avirulent bacteria *M*. *luteus* was used to infect female *L*. *migratoria*, and the fat bodies and ovaries of the locusts were dissected at 4 h and 6 d after treatment. Female locusts develop to sexual maturity at ~5 d after eclosion, and energy and resource accumulate during this period. Significant changes in fat bodies and in ovaries start at 7 to 10 d after eclosion, and varieties of ingredients in fat bodies and ovaries increased rapidly since 10 d [15]. Bacterial infection often elicits long-term changes in global host transcription. For example, flies that have survived from *M*. *luteus* infection remain chronically infected with ~2^10^ to 2^13^ bacteria per fly for at least 5.5 d [16]. Considering that the early response includes both an aggressive initial immune response and injury-induced transcriptional regulation [17], infected insects were collected at 144 h (6 d) after treatment, with 4 h after treatment used as the control time point.

GO analysis, which was used to explore the relationship between immunity and reproduction in locusts, indicated that GO terms related catalytic activity (n = 22) and response to oxidative stress (n = 18) were the most enriched. These biological functions are the basic response of the organism to external injury stimuli. Six GO terms associated with endosome metabolism were increased significantly. In eukaryotic cells, endosomes play a vital role in the regulation of fundamental processes, including nutrient uptake, immunity, signaling, adhesion, membrane turnover, and development, by regulating the re-utilization or degradation of membrane components. The up-regulation of functional groups associated with endosomes indicates an increase in metabolic waste emissions and cell activity in the fat bodies and ovaries [18]. The female locusts were at a stage where they were resisting bacterial infection and preparing for reproduction, which may have increased their metabolic activity. Lysosomes are single-membrane vesicles that release enzymes that degrade biological material [19] and, therefore, participate in waste removal. Four GO terms related to the JNK pathway, which regulates cell death in *Drosophila* and involved in the Toll pathway [20], were up-regulated, possibly indicating the occurrence of apoptosis in infected locust cells. GO terms for ‘innate immune response,’ ‘defense response,’ and ‘immune response’ were also up-regulated, thereby indicating that immune response of the infected locusts was heightened. Cellular immunity related GO terms, such as ‘autophagosome,’ ‘autophagy,’ and ‘regulation of autophagy,’ were significantly up-regulated, thereby indicating that the cells were fighting against pathogenic infection [21]. Three GO terms were involved in ovary development, including ‘ovarian follicle cell development’ (n = 23), ‘ovarian follicle cell stalk formation’ (n = 6), and ‘ovarian nurse cell to oocyte transport’ (n = 3). The MCM complex (n = 5) is a class of DNA replication genes. The JH-receptor complex has been reported to act on Mcm4 and Mcm7 to regulate DNA replication and polyploidy for vitellogenesis and oocyte maturation on migratory locusts [22]. The processes of immunity and reproduction are not separable from the nutrition metabolism of the body. Of the DEGs mapped to up-regulated GO terms, 158 and 38 were involved in immunity and reproduction, respectively, which suggested that more energy resources were allocated to bacterial defense.

The 1488 significantly altered transcripts were enriched in the KEGG pathway. The pathways related to immunity and reproduction was extracted to analyze trade-off strategies between the two processes. The top mapped pathways, namely ‘Endocytosis’ (n = 83), ‘Phagosome’ (n = 33), ‘Lysosome’ (n = 26), and ‘Regulation of autophagy pathway’ (n = 11), were involved in the elimination of infectious bacteria, viruses, and aging cells, as well as in the absorption of macromolecules in nutrient metabolism. Five DEGs were mapped to the Toll-like receptor signaling pathway, which is conserved among animals [23], and can be activated through gram-positive triggers [24]. In the present study, the *M*. *luteus* infection stimulated the immune response of the locusts, and the appropriate immune factors were up-regulated significantly. A total of 32 transcripts were mapped to ‘FoxO signaling pathway’. In the red flour beetle, the FoxO transcription factor can control vitellogenesis, thereby, reproduction by negatively regulating insulin-like peptide signaling, and FoxO can also activate AMPs independently of immune pathways [25]. The significant up-regulation of ‘Insulin resistance’ (n = 25) pathways promoted the FoxO signaling pathway [26] and inhibited nutritional metabolism, thereby reducing or even preventing oviposition [27]. These demonstrate that, at the biological pathway level, locusts allocate more energy into immune responses when faced with a trade-off between immunity and reproduction. Eighteen DEGs were mapped to the mTOR signaling pathway. It has been reported that the insulin-like peptide/TOR pathway plays a role in transducing nutritional information that regulates JH synthesis in mosquitoes and, thereby, determines the outcome of trade-offs between survival and reproduction [28]. The above analysis indicated that immune and reproductive processes are closely related to the nutritional status of individual locusts. ‘Insulin signaling pathway’ (n = 4) was up-regulated by infection. Combined with the sequence information provided by the NCBI and locust genome databases [29], *VgA* (LOCMI12128-m1) and *VgB* (LOCMI12129-m1) were found to be down-regulated (log_2_FC = -17.82 and -18.13, respectively). Vitellogenin is the precursor of vitelline which is the main egg-yolk protein in insects. It is mainly formed in fat bodies and is absorbed by oocytes through blood circulation [30]. Reduced vitellogenin expression in the fat body inhibits ovarian development and oocyte maturation. In the present study, Vg expression was significantly down-regulated, which suggests that locusts, when challenged by infection, may devote the majority of resources to maintaining humoral circulation and survival and reduce investment in reproduction. Insect defensins are effector components of the innate defense system. In locusts, there are four genes that code for defensins, namely LmDEF1, 3, 4, and 5 [31]. In the present study, *defensin 3* (LmDEF3, LOCMI01321-m1) was significantly up-regulated in the infected *M*. *luteus* group (log_2_FC = inf). The prophenoloxidase-activating cascade is one of the key components of arthropod immunity. Wounding or infection triggers the release of *PPO1* through a process that involves JNK activation [32]. In the present study, *PPO1* (LOCMI17352-m1) was up-regulated (log_2_FC = 5.93) in the infected group, which suggests that locusts are able to adapt to immune challenge. Furthermore, JH acid methyltransferase (LOCMI17613-m1) was also up-regulated (log_2_FC = 7.25) in infected group. This enzyme is involved in final steps of JH biosynthesis in insects [33], and it’s up-regulation directly increases JH expression. In turn, JH plays an important role in regulating Vg synthesis and oocyte maturation [22,34], as well as innate immunity. It has been reported in the mealworm beetle, *Tenebrio molitor* that reduce of phenoloxidase, a major humoral immune effector, was mediated by JH [35] and that JH is a hormonal immuno-suppressor [36]. At 6 d after emergence, locusts begin to store energy for reproduction. During this period, JH secretion increases normally, but in the infected locusts, it fails to promote enough Vg secretion. The up-regulation of AMPs and *PPO1*, the markers of humoral immunity, indicates that the body has invested a lot of energy resources into infection resistance by *M*. *luteus*. It is inferred that locusts, in the process of balancing resource allocation between immunity and reproduction, devote more resources to eliminating infectious bacteria and maintaining their own survival than to reproduction. The molecular mechanisms of locusts trade-off of energy allocation between immunity and reproduction were represented by differentially changed GO functional groups and biological pathways. The relationship of IIS, JH, the phenoloxidase cascade, and AMP secretion in locusts should be investigated further.

## Conclusion

In this study, transcriptome analysis was used to identify *M*. *luteus* infection on the transcription of locust ovaries and fat bodies during the reproductive preparation period. The differentially expressed transcripts indicated that the expression of *PPO1* and *defensin 3* increased significantly, whereas the expression of *Vgs* decreased significantly, and that locusts, when faced with the trade-off of immune response and reproduction, allocate most resources to the physiological process of resistance to infection. These results provide a foundation for further studies of the molecular mechanisms underlying immune and reproductive trade-offs in locusts and provide insight for the biological control of locusts.

## Supporting information

S1 Fig Lysosome pathway. Red indicates significantly up-regulated transcripts.
(JPG)

S2 Fig mTOR signaling pathway. Red indicates significantly up-regulated transcripts.
(JPG)

S1 Table qRT-PCR primer sequences. (XLS)

S2 Table Genes that were differentially expressed in infected and control locusts at 4 h after treatment. (XLS)

S3 Table Genes that were differentially expressed in infected and control locusts at 6 d after treatment. (XLS)

S4 Table Genes that were differentially expressed in control locusts at 4 h and 6 d after treatment. (XLS)

S5 Table Genes that were differentially expressed in infected locusts at 4 h and 6 d after treatment. (XLS)

S6 Table GO terms for genes that were differentially expressed in infected and control locusts at 6 d after treatment. (XLS)

S7 Table KEGG terms for genes that were differentially expressed in infected and control locusts at 6 d after treatment. (XLS)

S8 Table KEGG terms for genes that were differentially expressed in infected and control locusts at 4 h after infection. (XLS)

S9 Table KEGG terms for genes that were differentially expressed in the control group at 4 h and 6 d after infection. (XLS)

S10 Table KEGG terms for genes that were differentially expressed in the treated group at 4 h and 6 d after infection. (XLS)
