# Supplementary figures and images for: Transcriptome analysis of the molecular mechanism underlying immunity- and reproduction trade-off in Locusta migratoria infected by Micrococcus luteus
Source: PLoS One. 2019 Aug 14;14(8):e0211605. doi: 10.1371/journal.pone.0211605 (PMC6693777; doi:10.1371/journal.pone.0211605)

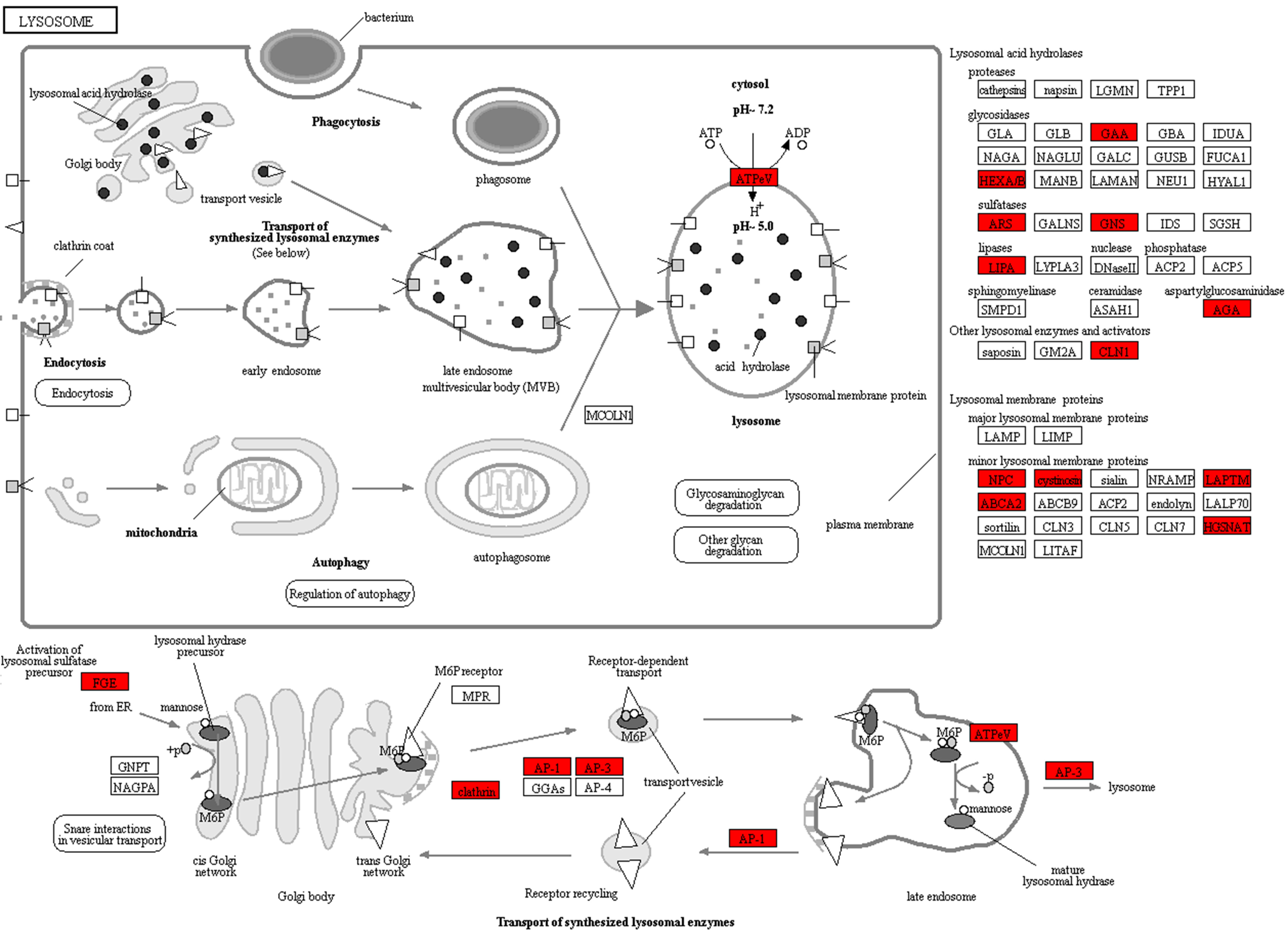

Supplement: S1 Fig — Red indicates significantly up-regulated transcripts. (JPG) [file pone.0211605.s001.jpg]

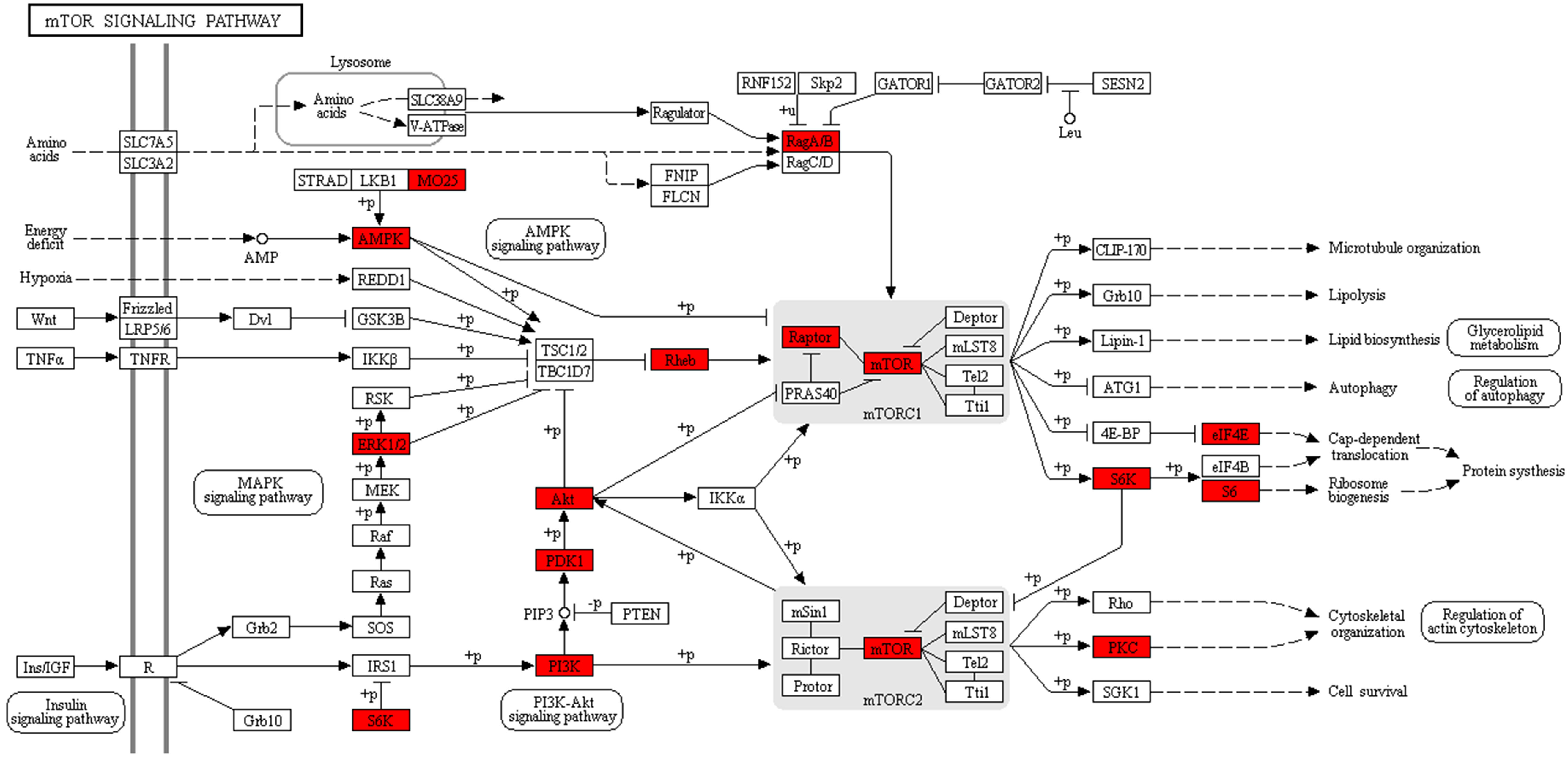

Supplement: S2 Fig — Red indicates significantly up-regulated transcripts. (JPG) [file pone.0211605.s002.jpg]
